# An alternative method for monitoring and interpreting influenza A in communities using wastewater surveillance

**DOI:** 10.3389/fpubh.2023.1141136

**Published:** 2023-07-27

**Authors:** Tomas de Melo, Golam Islam, Denina B. D. Simmons, Jean-Paul Desaulniers, Andrea E. Kirkwood

**Affiliations:** Faculty of Science, Ontario Tech University, Oshawa, ON, Canada

**Keywords:** influenza A, SARS-CoV-2, student absenteeism, wastewater, RT-qPCR

## Abstract

Seasonal influenza is an annual public health challenge that strains healthcare systems, yet population-level prevalence remains under-reported using standard clinical surveillance methods. Wastewater surveillance (WWS) of influenza A can allow for reliable flu surveillance within a community by leveraging existing severe acute respiratory syndrome coronavirus 2 (SARS-CoV-2) WWS networks regardless of the sample type (primary sludge vs. primary influent) using an RT-qPCR-based viral RNA detection method for both targets. Additionally, current influenza A outbreaks disproportionately affect the pediatric population. In this study, we show the utility of interpreting influenza A WWS data with elementary student absenteeism due to illness to selectively interpret disease spread in the pediatric population. Our results show that the highest statistically significant correlation (R_s_ = 0.96, *p* = 0.011) occurred between influenza A WWS data and elementary school absences due to illness. This correlation coefficient is notably higher than the correlations observed between influenza A WWS data and influenza A clinical case data (R_s_ = 0.79, *p* = 0.036). This method can be combined with a suite of pathogen data from wastewater to provide a robust system for determining the causative agents of diseases that are strongly symptomatic in children to infer pediatric outbreaks within communities.

## 1. Introduction

As the world continues to deal with ongoing challenges associated with the COVID-19 pandemic, the re-emergence of seasonal respiratory pathogens such as influenza poses an additive threat to public health. Influenza and pneumonia are ranked among the top 10 leading causes of death in Canada. It is estimated that influenza causes approximately 12,200 hospitalizations and 3,500 deaths per year (1). With numerous non-pharmaceutical interventions placed during the COVID-19 pandemic, the dynamics of influenza exposure and transmission, incidence rates, and symptom severity may have changed. This is evident by the current increase in influenza infections and influenza-associated hospitalization rates in Canada, which are above-expected levels that are typical for the flu season, spanning from August 2022 to February 2023 (1, 2). Thus, there is now an immediate demand for improved surveillance of this contagious disease.

Influenza viruses arise from the family Orthomyxoviridae. This family is unique in that they are enveloped viruses with genomes that consist of negative-sense single-stranded RNA segments (3). There are four types of influenza viruses, A, B, C, and D. Within these, influenza A is the only flu virus known to cause flu pandemics (4). The influenza A type virus is further classified into subtypes, clades, and subclades based on the presence of surface viral proteins hemagglutinin (H) and neuraminidase (N). Currently, the most commonly detected subtypes found circulating in human infections are A(H1N1) and A(H3N2) (4). The most commonly reported symptoms of the virus are fever, cough, runny nose, body aches, and sore throat (4).

One of the important lessons learned from the COVID-19 pandemic is that monitoring respiratory pathogens through conventional clinical testing (nasal swabs, sample collection, and RT-qPCR) presents many challenges and may not be sufficient for pathogen surveillance where timely information is required. Thus, the surveillance of pathogens in wastewater has been successfully implemented as a credible technique to complement monitoring SARS-CoV-2 infections within communities (4, 5). When clinical SARS-CoV-2 tests were widely available in Canada, they provided a reliable and robust metric that correlates with SARS-CoV-2 RNA concentrations in domestic wastewater (5, 6). However, interpreting SARS-CoV-2 wastewater data has been challenging (variable correlation strength, lack of reported cases, and inconsistent lead vs. lag association to clinical data). Additionally, public accessibility to clinical COVID-19 PCR tests has been greatly limited in Canada and is currently only available to high-risk groups.

Similar to current COVID-19 testing, influenza A testing is limited to people in hospitals or associated with an institutional outbreak (7). As such, there are incomplete incidence data available to compare with WWS viral signals, thus making the interpretation of wastewater epidemiology data very complex.

Wastewater monitoring is quickly emerging as a powerful epidemiological tool in public health surveillance and the early detection of contagious diseases. It is unbiased, inexpensive, and can be implemented easily, as one wastewater sample can be used to test small communities as well as large populations (8). In addition to SARS-CoV-2, wastewater surveillance can also be applied to target influenza and other pathogens using similar a DNA/RNA-based RT-qPCR detection methodology (9). For example, Mercier et al. (10) recently reported the feasibility of monitoring influenza A viral RNA gene copies in wastewater primary sludge within three distinct communities in Ottawa, Canada, with lead times between 14 and 21 days over clinical testing data.

In this study, we aimed to contribute to the growing WWS knowledge base by exploring other methodological approaches that aid in the interpretation of WWS data, particularly where the clinical case data are limited. Using a detection method focusing on primary influent, we explored the efficacy of school absences due to illness as a proxy measure of community influenza A prevalence and compared these inferred cases with influenza A viral loads in local domestic wastewater samples from Ajax, Ontario, Canada. This analysis will also allow the monitoring of influenza infections in the pediatric population, which likely serves as a major driver of total population influenza A prevalence in sewershed communities that flow into municipal wastewater treatment plants.

Using time-step Spearman's rank correlation analysis and pepper mild mottle virus (PMMoV) normalization to rescale influenza A and SARS-CoV-2 RNA gene copies in wastewater, we compared the relationships between levels of influenza A and SARS-CoV-2 gene copies and (1) student absences due to illness and (2) clinical cases of influenza A to determine the lead and lag time of influenza A WWS data using 1-, 3-, and 5-day averaging times.

## 2. Materials and methods

### 2.1. Wastewater sample collection and PEG-NaCl viral concentration

Raw wastewater samples were collected 3 days/week for almost 13 weeks from 15 September to 13 December 2022, from a sanitary sewershed pumping station in Ajax, Ontario, Canada that captures domestic wastewater from approximately 150,000 people. The sewershed primarily reflects a suburban residential area (>80%), with some commercial and light industries. Each sample represented hourly sub-samples of equal volume collected over a 24-h period, for a final composite sample volume of 500 mL that was stored at 4°C. Wastewater samples were transported in sterile, sealed 500 mL plastic containers at 4°C to Ontario Tech University, Oshawa, Ontario, Canada. Upon arrival, the samples were stored at 4°C for up to 24 h until processing and analysis.

To precipitate the influenza viral particles and PMMoV particles from wastewater, all samples were mixed thoroughly before 30 mL of wastewater was transferred to the Nalgene™ Oak Ridge High-Speed PPCO Centrifuge Tubes (Thermo Fisher Scientific, MA, USA) containing 10 mL of 4X PEG–NaCl buffer (40% w/v PEG 8,000 and 1.5 M NaCl), vortexed briefly and centrifuged using a SORVALL RC 6+ Ultracentrifuge (Thermo Fisher Scientific, MA, USA) at 12,000 x *g* for 2 h at 4°C (11, 12). After discarding the supernatant, a second centrifugation step at 12,000 x *g* for 10 min was performed to help solidify the pellet. The PEG–NaCl method was utilized for all experimental samples to concentrate the viral particles. Before RNA extraction, the pellet mass for all samples was measured using a top-loading balance (Sartorius, Goettingen, Germany).

### 2.2. Nucleic acid extraction

Total RNA was extracted from the concentrated wastewater pellets using the RNeasy^®^ PowerMicrobiome^®^ Kit (Qiagen, Germantown, MD) with the following alterations from the recommended protocol: 100 μL of phenol–chloroform–isoamyl alcohol (25:24:1, pH 6.5–8) was added to each sample prior to the lysis step (Thermo Fisher Scientific, MA, USA). The pellet was resuspended with 650 μL of the lysis buffer and transferred to the PowerBead (glass, 0.1 mm) tubes (QIAGEN, Germantown, MD). The subsequent steps were performed following the recommended protocol from the manufacturer's kit. The total RNA was eluted from the kit spin column using 100 μL of RNase-free water.

### 2.3. Quantitative reverse transcription PCR

Quantification of the influenza A matrix (M) gene, SARS-CoV-2 viral nucleocapsid (N) gene, and the PMMoV coat protein gene in the composite wastewater samples was performed using the Reliance One-Step Multiplex RT-qPCR Supermix (Bio-Rad, Hercules, CA) utilizing a TaqMan-MGB (Applied Biosystems, Massachusetts, USA) probe-based approach. Gene copy numbers of influenza A in wastewater were determined using the WHO influenza A M gene primer/probe to target a region of the M gene that encodes for the M1 protein. The gene copy numbers of SARS-CoV-2 in wastewater were determined using the US CDC 2019-nCoV N2 Assay RUO primer/probe mix to target a region of the N gene and have been discussed previously (13). PMMoV gene copy numbers were determined using PCR primers developed by Zhang et al. (14) to target a region of the PMMoV strain S genomic sequence. All probes/primers used in this study and their sequences are shown in Table 1.

**Table 1:** Listed are the primers and probes used to obtain WWS data.

**Viral target**	**Primer/Probe**	**Sequence (5′-> 3′)**	**References**
Influenza A	MP-39-67For	CCMAGGTCGAAACGTAYGTTCTCTCTATC	(33)
	MP-183-153Rev	TGACAGRATYGGTCTTGTCTTTAGCCAYTCCA	(33)
	MP-96-75ProbeAs	VIC-ATYTCGGCTTTGAGGGGGCCTG-MGBNFQ	(33)
SARS-CoV-2	2019-nCoV_N2 For	TTACAAACATTGGCCGCAAA	(34)
	2019-nCoV_N2 Rev	GCGCGACATTCCGAAGAA	(34)
	2019-nCoV_N2 Probe	FAM-ACAATTTGCCCCCAGCGCTTCAG-MGBNFQ	(34)
Pepper mild mottle virus (PMMoV)	PMMoV For	GAGTGGTTTGACCTTAACGTTGA	(14)
	PMMoV Rev	TTGTCGGTTGCAATGCAAGT	(14)
	PMMoV Probe	VIC-CCTACCGAAGCAAATG-MGBNFQ	(14)

Materials were obtained from Applied Biosystems (MA, USA). M = A/C, Y = C/T, R = G/A.

For each wastewater sample, technical replicates were run in triplicate, and serial dilutions of the Twist Synthetic H3N2 RNA Control (Twist Bioscience, CA, USA) were run on every plate to quantify the gene copies of influenza A (M gene) using the standard curve method. Each reaction comprised a mixture of 5 μL of RNA template, 600 nM (M1) of each forward and reverse primer, 100 nM (M1) probe, and 5 μL of 4X Reliance master mix for a final reaction volume of 20 μL. Reactions were performed in a CFX Connect Real-Time PCR Detection System (Bio-Rad, Hercules, CA) beginning with a reverse transcription (RT) step at 50°C for 10 min, followed by a polymerase activation at 95°C for 10 min, and then 45 cycles of denaturation and annealing/extension at 95°C for 10 s and then at 60°C for 45 s. The RT-qPCR analysis was validated with no-template controls (NTCs) using PCR grade water instead of RNA, no-reverse transcriptase controls (NRTs), and the presence of PCR inhibitors was determined using a serial dilution. All samples analyzed were quantified according to the MIQE recommendations (15) using the standard curve method with a synthetic RNA standard (Twist Synthetic H3N2 RNA Control, Catalog #: 103002) that contains the complete genome of influenza A/H3N2. A minimum 7-point standard curve with technical triplicates for each point was performed for every RT-qPCR experiment. The primer efficiency of influenza A (M1) was approximately 91%. The R^2^ value was ≥0.99, and the slope of the standard curve was ~3.55. The limit of detection for the influenza A M1 gene with a 95% coefficient of variation was 13.71 copies/mL of wastewater. Any crossing threshold values above 40 cycles were identified as negative reactions, assuming no amplification/detection occurred. The dynamic range of our linear standard curve was between 1 × 10^3^ copies/μL and 1.37 × 10^0^ copies/μL.

### 2.4. Influenza A case data

Influenza A case data for the city of Ajax were provided by the Durham Region Works and Health Department (DRHD) and represented cases identified within the sewershed when they were reported to DRHD.

### 2.5. School absences

DRHD also collected student absence data due to illness for all elementary and secondary schools within the region, as all schools are required to report absences due to illness. Within the region of Durham, the city of Ajax, ON, contains a total of 24 elementary schools (J.K.–grade 8) with approximately 13,500 students and a total of 3 secondary schools (grades 9–12) with approximately 6,000 students. The absenteeism data provided for this study did not include specific absenteeism for each school in Ajax, but rather a separate daily total percent (%) of absence due to illness (# of students absent due to illness/total student population ^*^ 100) for elementary and secondary schools. Absences due to illness were also collected for some Child Care Centers (CCC); however, these data were limited because CCC absence reporting was voluntary, and thus the sample size was too small for analysis.

The percentage of student absences due to illness obtained from DRHD is a measure of the cumulative prevalence of illness across schools (similar to the total number of cases). However, given that WWS captures the daily abundance of viral genes within the catchment area, for comparison purposes, we transformed the % school absence due to illness data to represent the changes in the daily incidence rate of illnesses in schools by calculating the overall percent change (daily % absence due to illness reported–% absence due to illness from the previous school day) for primary and secondary schools in Ajax. Positive percent change values represented the increase in daily incidence of illness within schools, while negative values represented a decrease in illness (students re-attending after recovery). Since we are not evaluating the effectiveness of non-pharmaceutical interventions, only positive percent changes in absences due to illness were used to infer the incidence of new cases.

### 2.6. PMMoV normalization for comparisons with influenza A case data and school absenteeism

Viral WWS data are normalized with PMMoV to account for the human fecal content in wastewater as PMMoV is generally found at consistent levels in wastewater (WW) and reflects population-level variability in waste production (14). PMMoV can also be used to account for variability caused by slight changes in extraction efficiency due to the complexity of the WW matrix and variability in pellet weight. This normalization approach is commonly used (10, 16–18) and helps not only reduce noise due to variability but also helps to scale the data for comparison with clinical surveillance data. Since PMMoV acts as a min–max normalization factor to scale the data, the maximum and minimum values are mostly within a 0 to 1 scale. This allows for comparison with other data from different time periods or even different sampling sites.

### 2.7. Statistical analysis

All data were assessed for normality. Wastewater viral concentrations and % change in absenteeism due to illness were not in compliance with parametric assumptions. Thus, a non-parametric Spearman's rank correlation coefficient (R_s_) analysis was performed using the daily PMMoV-normalized viral signals for influenza A: (1) the associated influenza A cases clinically reported and (2) the percentage (%) of change in school absenteeism for primary and secondary schools. To examine if the strength of associations between WWS data and clinical and absenteeism data can be improved with smoothed data, the correlation was also analyzed for 3-day and 5-day averages for both WWS data and the absence/case data.

In addition, to examine the maximum Spearman's correlation values, a time-step correlation analysis was conducted between WWS data and % change in school absenteeism and clinically reported cases with a data offset of a range of +/– 7 days applied to the % change in school absenteeism due to illness and reported cases time series data. This data shift in clinical and absenteeism metrics was applied to observe whether the correlation would be stronger with a lead (– shifted) or lag (+ shifted) time for up to 7 days. Zero-day offset refers to the correlation between wastewater signals and the case counts on the day of the wastewater sampling. Lead times refer to wastewater data being correlated with later case counts (e.g., a lead time of 3 days refers to the correlation between wastewater data and clinical cases 3 days later). Lag times refer to the wastewater data being correlated with earlier case counts (e.g., a lag time of 3 days refers to the correlation between wastewater data and clinical cases 3 days later). Corresponding *p*-values (obtained using the Mann–Whitney test) were also calculated to determine the statistical significance of each correlation (α = 0.05). Only *p*-values for strong correlation values (R_s_ > 0.50) are discussed below. For each averaging time, WWS data were only compared to absences/case data for the same averaging time. As per other studies (17) performing similar tests, the averages did not overlap, meaning it was not a moving average.

## 3. Results and discussion

### 3.1. Time-step correlation analysis between PMMoV-normalized WWS data and clinical surveillance metrics

**Figure 1:**
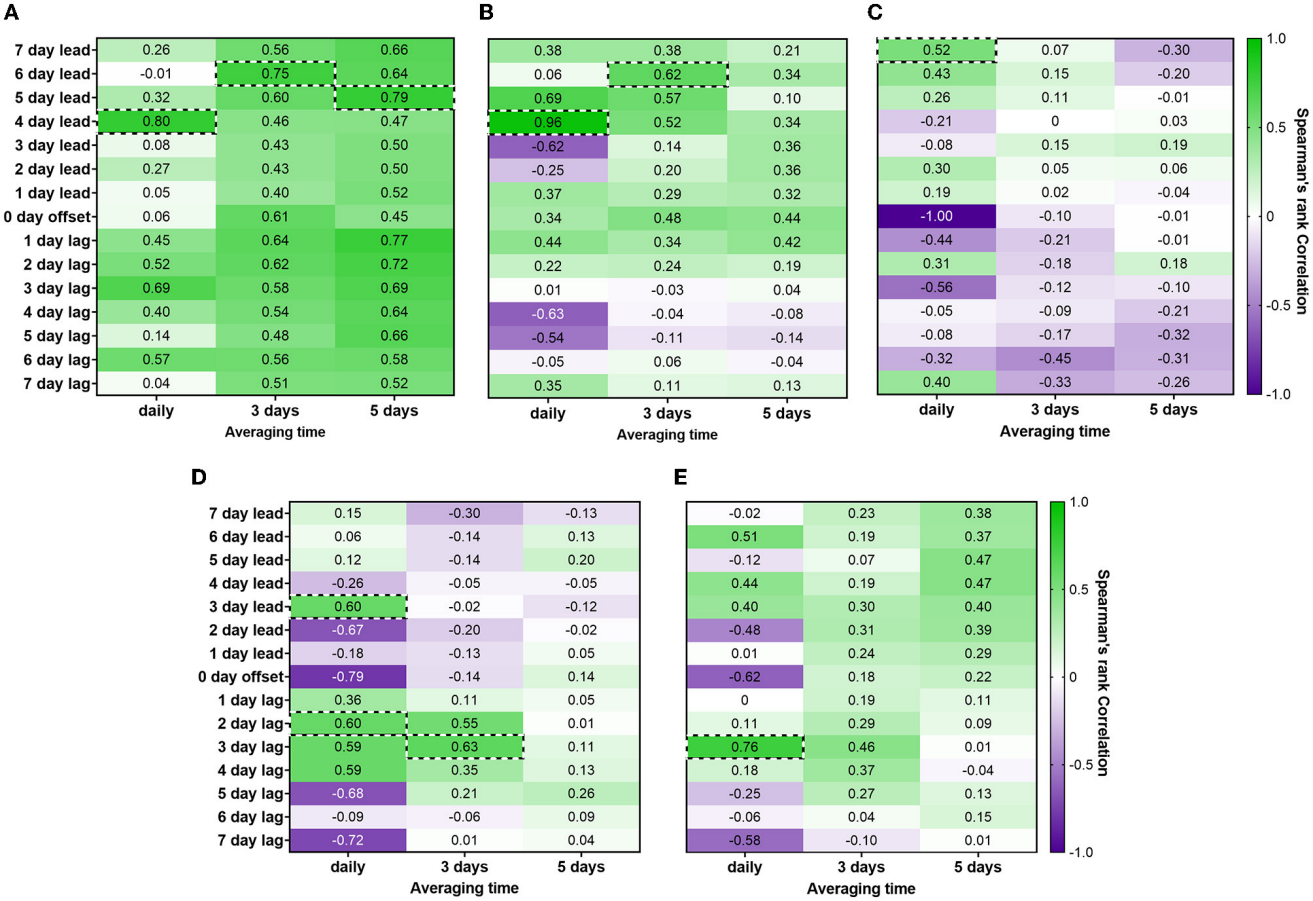
Results of time-step Spearman's rank (R_s_) correlation analysis with a 7-day lag to a 7-day lead. Maximum Spearman's rank correlation for each averaging time highlighted if R_s_ > 0.50 (strong correlation) or R_s_ > 0.30 but < 0.50 (moderate correlation) **(A)** PMMoV-normalized influenza A (M1 gene) viral signal vs. influenza A cases by reported date, **(B)** PMMoV-normalized influenza A (M1 gene) viral signal vs. % change in elementary school absences due to illness, **(C)** PMMoV-normalized influenza A (M1 gene) viral signal vs. % change in secondary school absences due to illness, **(D)** PMMoV-normalized SARS-CoV-2 (N2 gene) viral signal vs. % change in elementary school absences due to illness, and **(E)** PMMoV-normalized SARS-CoV-2 (N2 gene) viral signal vs. % change in secondary school absences due to illness.

Time-step correlation analyses were performed using PMMoV-normalized influenza A WWS data and cases of influenza A reported in the catchment area, which showed a strong correlation between influenza A WWS data and clinical surveillance data (Figure 1A). Comparisons of the daily PMMoV-normalized influenza A signal to the daily number of clinically reported cases showed a maximum Spearman's rank correlation coefficient value R_s_ = 0.80 (*p* = 0.579) when the data were adjusted with a 4-day lead time. Comparing the 3-day average for PMMoV-normalized influenza A WWS data to the 3-day average influenza A cases by reported date, the highest correlation (R_s_ = 0.75, *p* = 0.168) was observed with a 6-day lead time. However, these correlation values were not statistically significant. Only the 5-day average for PMMoV-normalized influenza A WWS data compared with the 5-day average influenza A cases demonstrated a strong significant correlation with clinically reported cases of influenza A (R_s_ = 0.79, *p* = 0.036) with a 5-day lead time (Figure 1A).

Time-step correlation analyses between PMMoV-normalized WWS data and clinical surveillance metrics have been previously explored and shown to effectively determine a lead time for COVID-19 WWS surveillance data (8, 17–23). Although, many have stated that the differences in gastrointestinal replication and fecal shedding of SARS-CoV-2 and influenza A were a cause for concern with respect to the effective detection[/interpretation] of influenza A in wastewater (12, 24–28). Our study has demonstrated that a 5-day lead time between smoothed datasets (5-day averaged influenza A WWS data and 5-day averaged influenza A cases) provided a strong significant correlation (R_s_ = 0.79, *p* = 0.036), indicating the presence of influenza genes in wastewater was found 5 days before the increase in clinically reported influenza cases.

This successful detection of influenza in raw influent wastewater and its correlation to clinical cases complements other recent studies (10, 25) that have also documented successful influenza A detection in both influent and sludge samples. Researchers examining primary sludge from communities in Ottawa were able to detect influenza A with a 14–21-day lead time against reported clinical case data (10). We were unable to detect influenza A in wastewater prior to the first identified case of influenza A within the catchment. However, this is unsurprising given the differences in the viral abundance of enveloped viruses that have been identified between primary sludge and primary influent (17, 21, 29, 30).

### 3.2. Correlation between PMMoV-normalized viral WWS data and % change in absences due to illness in elementary and secondary schools

Examining the correlation between the daily PMMoV-normalized influenza A WWS signal and the daily percentage of change in elementary school absences (see Figure 1B), the time-step correlation analysis showed that the maximum significant correlation value was obtained with a 4-day lead time (R_s_ = 0.96, *p* = 0.011). Comparisons of the smoothed 3-day averages of influenza A WWS data and % change in elementary school absences due to illness produced only a weaker correlation with a 6-day lead time (R_s_ = 0.62, *p* = 0.035), while no correlation (R_s_ < 0.5) was observed with the 5-day averaged dataset. The correlation of daily WWS data to primary school absenteeism (R_s_ = 0.96) with a 4-day lead time was much higher than associations with clinically reported cases (R_s_ = 0.79, *p* = 0.036) with a 5-day lead time.

In terms of the correlation between influenza A WWS data and the daily % change in absences due to illness in secondary schools (see Figure 1C), the time-step correlation analysis demonstrated a weak significant correlation with a 7-day lead time (R_s_ = 0.52, *p* = 0.011). Moreover, weak correlations (R_s_ < 0.50) were observed when averaging the data across 3 and 5 days.

We also concurrently monitored for the presence of SARS-CoV-2 RNA viral signal in wastewater from the same samples. This demonstrated that our experimental method can be utilized to detect both SARS-CoV-2 and influenza in wastewater influent. For elementary schools, only the correlations between the daily PMMoV-normalized SARS-CoV-2 WWS data and the daily % change in elementary absences due to illness showed a moderate significant correlation (R_s_ = 0.60, *p* = 0.017) with a 3-day lead and 2-day lag time of WWS data. However, in contrast to influenza A, the daily PMMoV-normalized SARS-CoV-2 WWS data showed a stronger statistically significant correlation (Rs = 0.76, *p* = 0.005) with the % change in secondary school absences due to illness, with a 3-day lag time (see Figure 1D).

When comparing the correlations between influenza A WWS data and % change in absences due to illness for elementary and secondary schools, the maximum Spearman's rank correlation coefficients were observed when looking at elementary absences (% change) due to illness. For each data set, regardless of the daily, 3-, or 5-day averages, elementary school absenteeism was observed to correlate significantly higher with influenza A WWS data than secondary school absences. This suggested that influenza A was potentially a causative agent in the absences of the elementary school students in the studied sewershed. Conversely, a strong and significant correlation was found between SARS-CoV-2 WWS data and secondary school absences (% change) due to illness. This may be due to the notable differences in disease presentation between influenza A and COVID-19 in children, where the former is commonly symptomatic compared to the latter (31, 32). However, due to the limited data, we could not confirm the number of SARS-CoV-2 cases in secondary school students (14–18 years old) to corroborate our findings.

### 3.3. PMMoV-normalized viral WWS data trends over time

**Figure 2:**
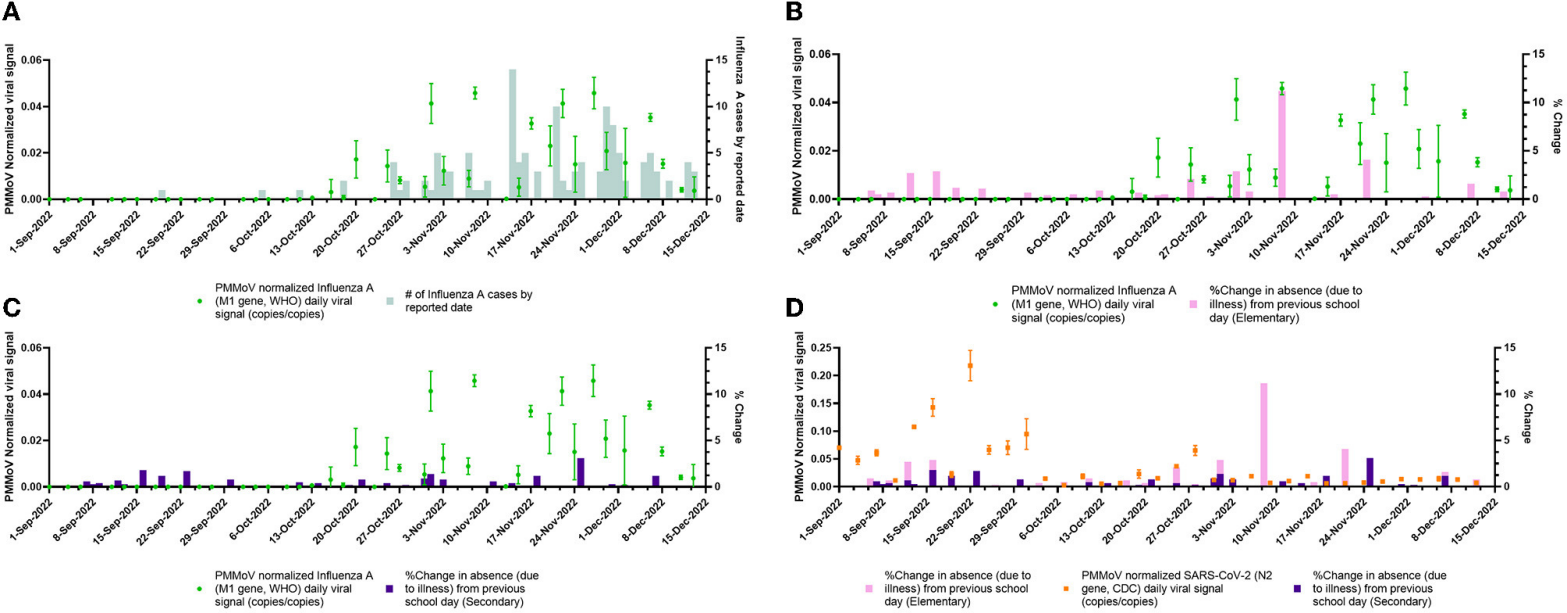
PMMoV-normalized viral wastewater signals vs. clinical surveillance metrics over the study period (1 September 2022 to 13 December 2022). **(A)** daily PMMoV-normalized influenza A (M1 gene, WHO) viral signal plotted with the number of new influenza A cases by reported date. **(B)** Daily PMMoV-normalized influenza A (M1 gene, WHO) viral signal plotted with daily % change in elementary school absences due to unspecified illness. **(C)** Daily averaged PMMoV-normalized influenza A (M1 gene, WHO) viral signal plotted with daily % change in secondary school absences due to unspecified illness. **(D)** Daily PMMoV-normalized SARS-CoV-2 (N2 gene, CDC) viral signal plotted with daily % change in elementary and secondary school absences due to unspecified illness.

The monitoring of PMMoV-normalized influenza and SARS-CoV-2 viral signals over time is shown in Figure 2. An increase in influenza WW signal can be observed from 13 October 2022 to 13 December 2022 along with increasing numbers of new influenza cases reported within that time period which demonstrated that WWS data may have an equivalent predictive power as clinical testing. The PMMoV-normalized influenza wastewater signal to % change due to absenteeism in elementary and secondary school is also shown in Figures 2B, C. Thus, wastewater surveillance was successfully employed using primary influent samples and identified the influenza A outbreak within the community.

## 4. Conclusion

This study confirms that a primary influent-based wastewater surveillance method is effective at monitoring influenza viral loads in wastewater and that it can be monitored concurrently with other infectious viruses such as SARS-CoV-2 using the same viral RNA concentration and RT-qPCR method for both targets. Additionally, this study demonstrated that school absenteeism may be a useful tool for interpreting influenza A disease prevalence within a pediatric population, and by extension, the total population within a given sewershed.

Our results show that the highest statistically significant correlation (R_s_ = 0.96, *p* = 0.011) occurred between daily influenza A WWS data and elementary school absences due to illness. This correlation coefficient is notably higher than the highest statistically significant correlations observed between influenza A WWS data and influenza A clinical case data (R_s_ = 0.79, *p* = 0.036). Correlations between influenza A WWS data and absences in secondary school were the lowest overall (see Figure 1C). Interestingly, SARS-CoV-2 showed contrasting results compared to influenza A WWS data, and the highest statistically significant correlation observed was between SARS-CoV-2 WWS data and secondary school absences (R_s_ = 0.76, *p* = 0.005). While SARS-CoV-2 WWS data and elementary absences showed inconclusive results.

While absenteeism is a more coarse metric and relatively ambiguous compared to clinical data, absences are less influenced by sampling bias than clinical tests. This sampling bias is due to clinical tests being reserved for a relatively small subset of the population (typically the elderly or young children) that elect to seek a healthcare intervention, whereas school absences are legally required to be reported to the school by caregivers.

Overall, our results show great promise for inferring influenza A prevalence in sewage-surveilled communities by adding student absenteeism to the wastewater epidemiologist's toolbox. However, the application of this tool comes with some advantages and disadvantages. With respect to advantages, we have confirmed that there is a strong correlative relationship between specific clinical indicators (influenza A cases) and WWS data. In addition, we found an even stronger correlative relationship between a non-specific clinical indicator (% change in elementary absences due to illness) and WWS data. However, this method of comparing non-specific clinical indicators of the pediatric population with WWS data could be further improved by supplementing with more WWS data representing other clinically significant pathogens circulating within the pediatric population such as respiratory syncytial virus (RSV). For example, RSV also has a notable symptomatic presentation in pediatric populations compared with older age children. Another potential limitation with this approach may be that the WWS data are impacted by “legacy viruses” that remain pseudo-persistent in wastewater and are later resuspended under high flow or other turbulence events and detected at higher concentrations using qPCR. However, the fate and stability of viruses in wastewater have not yet been determined. Introducing an effective sampling strategy where sampling sites are carefully selected and composite sampling is utilized with a higher sampling frequency can increase the chances of monitoring for “legacy viruses” due to resuspension or sloughing events. The success of SARS-CoV-2 surveillance programs worldwide has demonstrated that WWTP from different countries, populations, catchment sizes, and designs, can all be sampled and provide a very strong estimation of COVID-19 prevalence without being affected by legacy virus concentrations (6, 26, 35). This WWS method for the detection of influenza is not capable of predicting or forecasting the number of students absent due to a specific pathogen. However, this method, combined with a suite of pathogen data from WWS, is reasonable enough to provide a robust system for determining the causative agents of diseases that are strongly symptomatic in children to infer pediatric outbreaks. This kind of information could then be used to inform public health interventions aimed at pediatric populations as well as the larger community.

## Data availability statement

The raw data supporting the conclusions of this article will be made available by the authors, without undue reservation.

## Author contributions

TM conceived the study, performed the lab work, generated data, performed data analysis/interpretation, and wrote and reviewed the finished paper. GI contributed to the study concept and design, data interpretation, and writing and review. DS contributed to the study concept and design, data interpretation, and funding. J-PD contributed to the study concept and design, data interpretation, writing and review, and funding. AK contributed to the study concept and design, statistical analyses, data interpretation, writing and review, and funding. All authors contributed to the article and approved the submitted version.
